# Event and Comment

**Published:** 1913-01-15

**Authors:** 


### DENTAL REGISTER.

Vol. LXVII. January 15, 1913. No. 1

### EVENT AND COMMENT.

COLLEGE ADVERTISING. In looking into the new edition of Polk's Dental Register, we opened to a certain page on which appeared in conspicuous display type the advertisement of a dental college, with the following "catch line," "*A school of Dentistry, by Dentists for Dentists.*" After enumerating its virtues the advertisement closes with this sentence: "It is the only school in the State teaching a graded course in compliance with the rule of the Dental Faculties Association." Knowing there were two other colleges in the same city we were curious to know what they had to say for themselves. The next one had the same amount of space, a half page with a conspicuous display advertisement also, the first line of which set forth in big black type, this statement: "Today you should write for the big free catalog of the ——'s LEADING DENTAL COLLEGE." The third school likewise had its half page ad with this praiseworthy (?) statement: "The only Dental College in the State owning its own buildings. Has a complete equipment and competent corps of seventeen teachers." Now what we are wondering about is how a poor ignorant freshman gets into the right place, or at least feels satisfied with the choice he has made when he matriculates in either of these schools. Then again we are wondering if the Deans of these three schools who sign these three advertisements would like to see such advertisements printed alongside of each other in the daily papers of their city calling the attention of the public to their respective merits as professional servants. We don't believe it pays or is at all necessary.

PROFESSION OR TRADE. The following quotation from an editorial in the Long Island Medical Journal by Dr. Jacobson is of exceeding interest, and ought to be carefully considered by some of our Dental Editors who are turning much of their time and space into the "practice building" propaganda.

Look first on this picture: "Early in your practice get in the habit of regarding your every sentence like the grocer does his sugar or tea, or an artisan does his time." Dr. T. F. Reilly, in *New York Medical Journal.* In other words barter your skill and serve by the clock.

And then on this: "Our forefathers in the healing art were afflicted with a false modesty which made them ashamed to take money. The healthy way to look upon one's business position is this: I am not half paid for all the good I do. A doctor must be either a business man or a beggar." Dr. Brady in *N. Y. State Journal of Medicine.*

And thirdly on this: There is not, there never has been, a profession equal to ours, when practiced as it ever should be with a soule above lucre. Let us say and believe truly, that we entered the profession, not for the loaves and fishes, but to serve God and man in the best and highest way. To every physician who has the nobility of his profession at heart, the sending of his bill for services to a patient is always somewhat of a regret." Dr. Robinson in *N. Y. State Journal of Medicine.*

Problem: Find the most lovable man and the best doctor.

The moral of this recital is not so difficult to comprehend as the situation that confronts all professional men. How shall professional men of all sorts dignify their callings on the ideal basis of enlightened service to the needy on the altruistic doctrine,— and live as we are expected to do? There can be no question that the doctor, dentist or lawyer, who gives himself up body and soul to acquiring the knowledge and skill necessary for successful work in his field of endeavor will sooner or later, realize that his is a calling of the noblest sort, just as the earnest and faithful preacher thinks of his work. If he can do so he will get so much pleasure out of successfully relieving the discomforts of others that he may and often does become careless of his commercial relations. We wish there were a way out of this financial care for him, that he might be free to do more freely for others and that he might thoroughly enjoy loosing himself as the poet or artist does in the work that means everything worth while in life to him. But how can he do it? And the worst of it all is, in so far as he can not loose himself in his real work just so far he is liable to be dissatisfied with himself, and he will fail of that greater influence on human society which he should exercise. If Rockefeller, Morgan and Carnegie, could only come to the rescue; or the National Government should realize that money spent in providing professional service to its people will hasten the coming of Peace on earth more than all the armies and navies that will ever come into existence, we could hope for the realization of a class of ideal professional servants. Since we can't change conditions we shall need to hold our ideals so tenaciously as not to permit the undue influence of commercialism.

Lets be frugal business men if necessary, but make it always subordinate to the greater goal of benevolent professionalism. If we can do both we shall be greater and more independent perhaps. We certainly can not afford to put our careers on the basis of the bargain counter. Can there be such a thing as a consecrated life for professional callings?

MEDICAL PROFESSIONS TROUBLES. There is not only a spirit of unrest, but much sharp comment in the medical press of the country, not entirely due to contentions of the different sects, but in several other directions. The growth and development of municipal medical charities, and privately endowed hospitals has long been frowned upon as an encroachment on the privileges of the practitioner. Now it is the methods of conducting them that is coming in for criticism. An eminent Austrian who recently visited this country has criticised the fact that we loose the scientific interest and value of our hospitals because we make little investigation of the post mortem products. This criticism is unjust perhaps when it refers to hospitals maintained particularly for the instruction of medical students; but the large majority of our hospitals are patronized by patients who pay for treatment and will not submit to such demonstrations. A proposal is now being discussed of extending the medical course of study another year and making it wholly a hospital course. The trouble will be to make facilities to accommodate all the medical students in existing hospitals. The suggestion is made that the free wards in municipal hospitals shall be so utilized and that these patients be used in the training classes. If the corps of instructors can be made up of skilled men who can be paid enough to enable them to give all their time to the work it will succeed; otherwise the people will hardly submit to such an arrangement.

ROOT FILLING. We print in this issue a well studied article on filling root canals for permanent and sterile results with paraffin. The article is well worth every ones careful attention, as it may lead to safer method of accomplishing this important operation.

